# Factors Influencing the Intention of Perinatal Nurses to Adopt the Baby-Friendly Hospital Initiative in Southeastern Quebec, Canada: Implications for Practice

**DOI:** 10.1155/2014/603964

**Published:** 2014-07-02

**Authors:** Guylaine Chabot, Marie Lacombe

**Affiliations:** ^1^Evaluation Platform on Obesity Prevention, Quebec Heart and Lung University Institute, 2725 Chemin Ste-Foy, Local Y4283, Québec, QC, Canada G1V 4G5; ^2^Nursing Sciences, University of Quebec in Rimouski, Campus de Lévis, 1595, Boulevard Alphonse-Desjardins, Local 3056, Lévis, QC, Canada G6V 0A6

## Abstract

Nurses play a major role in promoting the baby-friendly hospital initiative (BFHI), yet the adoption of this initiative by nurses remains a challenge in many countries, despite evidences of its positive impacts on breastfeeding outcomes. The aim of this study was to identify the factors influencing perinatal nurses to adopt the BFHI in their practice. *Methods*. A sample of 159 perinatal nurses from six hospital-based maternity centers completed a survey based on the theory of planned behavior. Hierarchical multiple linear regression analyses were performed to assess the relationship between key independent variables and nurses' intention to adopt the BFHI in their practice. A discriminant analysis of nurses' beliefs helped identify the targets of actions to foster the adoption the BFHI among nurses. *Results*. The participants are mainly influenced by factors pertaining to their perceived capacity to overcome the strict criteria of the BFHI, the mothers' approval of a nursing practice based on the BFHI, and the antenatal preparation of the mothers. *Conclusions*. This study provides theory-based evidence for the development of effective interventions aimed at promoting the adoption of the BFHI in nurses' practice.

## 1. Introduction

Exclusive breastfeeding during the first six months of life is vital to the short and long-term health of the baby. Nevertheless, rates are still low in both developing and developed countries [1]. Breastfeeding is also beneficial for maternal health [2, 3] and is financially sustainable for the health system [4–6]. In 1991, the WHO/UNICEF launched the BFHI to promote exclusive breastfeeding in maternity units. The BFHI relies on the implementation of the Ten Steps to Successful Breastfeeding (Table 1) and the compliance to the International Code of Marketing of Breast-milk Substitutes (CODE). The CODE is a global public health strategy recommending restrictions on the marketing of breast-milk substitutes, such as infant formula [7]. Additionally, the hospital must achieve a 75% rate of exclusive breastfeeding and successfully pass an external accreditation process [8]. A systematic review found a statistically significant increase in breastfeeding initiation and exclusive breastfeeding at six months postpartum following the implementation of structured breastfeeding programs such as the BFHI [9]. Other studies reported an association between the BFHI and longer breastfeeding duration and exclusivity [10, 11], as well as improved breastfeeding self-efficacy at four weeks postpartum [12]. Implementation of the baby-friendly initiative in the hospital and in the community is also associated with improvements in health professionals' skills, knowledge, and attitude towards the conditions to successful breastfeeding [13]. The BFHI is the most widely promoted initiative for increasing breastfeeding rates [14]. Currently, there are approximately 22,000 baby-friendly hospitals in 157 countries [1].

In Canada, maternity centers have substantially improved their implementation of the BFHI since 1993 [15]. In 2012, the national breastfeeding initiation rate was 90.3% and the exclusive breastfeeding rate was 24.2% at six months postpartum. The initiation rate for the Province of Quebec corresponds to the national average and shows leveling off since 2008 (89.9%) and the exclusive breastfeeding rate at six months is the lowest (16.1%) in Canada [16]. The Quebec Ministry of Health and Social Services has chosen the BFHI as the main strategy to promote exclusive breastfeeding in 2001 [17], but regional disparities with breastfeeding practices are noteworthy [18]. Hospital-based maternity units located in South-Eastern Quebec have been trying for nearly a decade to obtain their BFHI accreditation without success. Hospital administrations noticed a mitigated enthusiasm among perinatal nurses. Their concern was brought to the attention to the Regional Health Agency Breastfeeding Branch, which contacted the researchers to study the motivation of these nurses to adopt the BFHI in their practice and identify potential solutions for its successful implementation.

Studies dedicated to understanding the factors motivating perinatal nurses to adopt the BFHI in their practice are still scarce and often anecdotal. In addition, among studies exploring factors related to the adoption of the BFHI in nursing practices, it can be challenging to identify which factors to prioritize to inform interventions to foster the adoption of the BFHI at the nurses' individual level [17]. Nurses play a central role in promoting the BFHI [19], yet the successful adoption and implementation of the BFHI by nurses remain a challenge in many countries, even in “BFHI accredited” hospitals and the ones that successfully met all the required conditions [4, 11, 20, 21]. Therefore, the aim of this study was to identify the factors influencing the intention of hospital-based perinatal nurses to adopt the BFHI in their practice.

## 2. Theoretical Foundations

The perspective of healthcare professionals is crucial to their adoption of clinical practices [22]. An extended version of the theory of planned behavior (TPB) [23] was used to guide this study. TPB is known for its capacity to explain and predict the intention of healthcare professionals to adopt clinical practices, especially among nurses [22, 24]. In addition, TPB takes into consideration behavior not completely under volitional control of individuals, which is the case with the participants who are subject to the rules of their hospitals. TPB encompasses four constructs:* intention, attitude*,* subjective norm*, and* perceived behavioral control*. Ajzen's theory holds that* intention* and* perceived behavioral control* (PBC) are direct predictors of healthcare professional practices.* Intention* corresponds to the motivation to perform a given practice.* PBC* refers to the degree of ease or difficulty with which clinical practices can be adopted [23]. In turn,* intention* is predicted by* attitude*,* subjective norm,* and* PBC.* In this study,* attitude* refers to a more or less favorable evaluation of the adoption of the BFHI.* Subjective norm* (SN) corresponds to nurses' perception of approval by referent individuals (e.g., colleagues, mothers).* Attitude*,* SN*, and* PBC* are, respectively, defined by behavioral, normative, and control beliefs. The behavioral beliefs refer to the perceived advantages and disadvantages of adopting the BFHI. The normative beliefs reflect the perceived degree of approval of the adoption of the BFHI. The control beliefs are the barriers and facilitators to the adoption of the BFHI. TPB has been used to study breastfeeding intention among women in multiple contexts [25–27], as well as nurses and dieticians' intention to recommend breastfeeding to mothers during the first six months after birth [28]. To our knowledge, the present study is the first to rely on Ajzen's theory to explain the underlying mechanisms motivating nurses to adopt the BFHI in their practice. In order to gain a global understanding of the studied nursing clinical practice, Perkins et al. [29] recommend the addition of psychosocial constructs to the TPB model. Godin and colleagues' [22] systematic review revealed that the addition of variables such as Triandis's moral norm contributed to higher value of the explained variance of the intention of health professional. Therefore,* moral norm* and the past adoption of a practice (*past behavior*) borrowed from the theory of interpersonal behavior [30] were added to the TPB model.* Moral norm* takes into consideration the ethical dimension of healthcare professionals' practice and provides information on the moral obligation felt by the participants to adopt clinical practices [22].* Past behavior* was measured as past experiences can affect the perceived control of a situation [31]. This version of the TPB explained 83% of the intention of school nurses to adopt a new role [32].

## 3. Methods

### 3.1. Design and Participants

This cross-sectional study relied on a survey. The participants under study were perinatal nurses who dispense care to pregnant women from admission to discharge. Nurses meeting the inclusion criteria (*n* = 241), which consisted of having a part-time or full-time position in the maternity unit with a minimum of 30 shifts within the previous six months, were solicited from the six hospital-based maternity units located in southeastern Quebec. Among nurses solicited, 160 accepted to participate and 159 (66%) successfully completed the survey. One questionnaire was rejected due to missing data. Rashidian et al. [31] recommend a sample size of 148 to predict the intention based on the TPB models.

### 3.2. Data Collection

Data collection took place in the maternity units during fall 2011 and winter 2012. Each hospital administration designated a local contact person who met all nurses in order to explain the project and to distribute the information pamphlet, the consent form, and the questionnaire to them. Potential participants were invited to participate to the interviews, the test-retest, and the survey. Two different groups of nurses participated to the interview and the test-retest. However, some nurses contributed both to the interview and to the survey when they indicated their willingness to participate in both processes on the consent form. The number of nurses who participated in the interview and the test-retest were proportionally distributed across maternity units in order to represent the total number of perinatal nurses in each unit. Potential participants had two weeks to complete and return the coded questionnaire anonymously to the researchers. The study was approved by the ethic committees of the University affiliated hospital (CHA-Hôtel-Dieu de Lévis) and the University of Quebec in Rimouski. All procedures followed were in accord with the ethical standards of the responsible institutional committees.

#### 3.2.1. Instrument Development

The BFHI Steps (3 to 10) were presented at the beginning of the questionnaire. Nurses were not surveyed on Steps 1 and 2 (see Table 1), which are organizational measures that are often beyond the control of the individual care provider. The questionnaire was developed according to Ajzen's [33] guidelines for developing a TPB questionnaire, which is composed of belief-based items obtained from interviews, as well as items measuring theoretical constructs. To identify nurses' beliefs on their intention to adopt the BFHI in their practice, individual semistructured telephone interviews were conducted with a sample of 25 perinatal nurses representing the maternity units under study. A total of six questions dealing with nurses' perceived pros and cons of adopting the BFHI, barriers, and facilitating factors and individuals or groups favorable or unfavorable to the adoption of the BFHI in their practice were developed. The interviews were audiotaped and lasted 20 to 25 minutes. The content analysis was performed independently by three researchers who agreed on the classification and labeling of themes extracted. To identify the nurses' salient beliefs, a frequency of mention was established for each category and those most frequently mentioned (up to 75% of the total) were added to the questionnaire to form the belief-based items.

The items measuring the theoretical constructs (*intention, attitude, PBC, SN, moral norm, and past behavior*) were developed as follows:* intention* was measured with three items, such as “…I am determined to adopt Steps  3 to 10 of the BFHI in my practice” (seven-point scale: 7 = strongly agree; 1 = strongly disagree). Five items served to measure* attitude*: “To adopt Steps  3 to 10 in my practice would be…” (seven-point scale: 7 = very useful; 1 = very unuseful).* Perceived behavioral control* was measured with four items, such as “I would find difficult to adopt Steps  3 to 10 of the BFHI in my practice” (seven-point scale: 7 = strongly agree; 1 = strongly disagree). Three items were used to measure* subjective norm*: “My professional and personal entourage think I should adopt the Steps 3 to 10 of the BFHI in my practice” (seven-point scale: 7 = strongly agree; 1 = strongly disagree). Three items measured* moral norm*: “… I would feel guilty if I was not adopting Steps 3 to 10 in my practice.” Eight items measured* behavioral beliefs*, such as “To adopt Steps 3 to 10 in my practice would increase my workload” (seven-point scale: 7 = strongly agree; 1 = strongly disagree).* Facilitating factors* were measured with five items starting with “I would adopt Steps 3 to 10 in my practice if…” followed by, for example, “…there was a lactation consultant in the maternity unit” (seven-point scale: 7 = strongly agree; 1 = strongly disagree). Potential* barriers* were measured with four items, such as “I would adopt Steps 3 to 10 in my practice, despite “… the lack of motivation of the mothers towards breastfeeding”” (seven-point scale: 7 = strongly agree; 1 = strongly disagree).* Normative beliefs* were measured with six items as follows: “If I adopted Steps 3 to 10 in my practice, the following persons would approve/disapprove…” (seven-point scale: 7 = strongly approve; 1 = strongly disapprove). Finally, one item measured* past behavior*.

The questionnaire was pilot tested with a group of seven experts in psychosocial theories and a group of five nurses (a lactation consultant, a regional maternity health care advisor, and three perinatal nurses from participating hospitals) for clarity and readability.

#### 3.2.2. Psychometric Qualities

The reliability of the questionnaire was assessed by a test-retest with a total of 26 nurses from the units under study who completed the same version of the questionnaire twice at two-week intervals. The internal consistency was assessed by means of Cronbach's alpha coefficient [34]; the values varied between 0.60 and 0.92. The temporal stability assessed by means of the intraclass correlation coefficient yielded values varying between 0.52 and 0.84, which represent moderate to good coefficients of agreement [35]. The final instrument consisted of 55 items, including demographic variables. The internal consistency of the variables varied between 0.66 and 0.90, which is considered satisfactory for an exploratory study [36].

### 3.3. Statistical Analyses

Descriptive analyses of the sample of nurses were first performed (means and standard deviations), followed by a correlational analysis with Pearson or Spearman coefficients between studied variables. Subsequently, the independent variables statistically correlated with* intention* were entered in the hierarchical multiple linear regression analysis to identify the determinants of the intention of nurses to adopt the BFHI in their practice. The TPB constructs were first entered, followed by* moral norm*,* past behavior*, facilitating factors, barriers, normative beliefs, behavioral beliefs, and the demographic characteristics of the participants, respectively. A chi-square test of the maternity units was also performed. Based on the smallest number of steps (BFHI) fully implemented, maternity unit 1 was chosen as the reference organization [37]. Maternity units were coded and entered last in the model (as dummy variables). After each step, variables not reaching the minimum threshold (*P* < 0.05) were excluded from subsequent steps. von Haeften et al. [38] suggest that an intervention should attempt to change the underlying beliefs carrying the strongest relative weight as predictors of* intention*. In order to identify the beliefs that could serve to guide relevant decisions, a discriminant analysis contrasting high and low intenders was performed. All analyses were performed using SAS software, version 9.2 [39].

## 4. Results

### 4.1. Descriptive

Participants were all females. The majority held a college diploma, a full-time position, had more than one year experience in perinatal care, and had received a 2-hour training on breastfeeding within the past five years. Due to the importance of missing data (greater than 50%), the following demographic items were rejected: “Did you breastfeed your children?” and “Globally, how would you qualify your breastfeeding experience?” Descriptive statistics of the sample of nurses are presented in Table 2.  Table 3 presents a portrait of the maternity units showing that maternity units 2 and 3 have successfully implemented most of the steps, including the WHO/UNICEF 20-hour training, with an additional hour dedicated to breastfeeding skills (Step 2). These two units are the only ones having a local lactation consultant and recording data on exclusive breastfeeding at discharge. These units also have a breastfeeding policy (Step 1). The maternity unit that implemented the most steps has the best ratio of maternity nurses per birth volume yearly. Nonetheless, according to data communicated by the contact persons of each maternity unit, none of their organization has yet successfully implemented Step 6 (80% of the time based on the responses of mothers randomly selected in a hospital self-assessment survey). Each unit has a committee that monitors the implementation of the BFHI. The standards for the accreditation may vary across centers, according to their mission (regional or local), for instance. The monitoring process of the implementation of the steps and the rate of exclusive breastfeeding were not always monitored with standard procedures in four of the six units. Only two units used a standardized form, the WHO BFHI self-assessment survey [8], to assess the implementation of the BFHI. Therefore, the information provided regarding the implementation of the steps might not accurately represent the state of implementation in these units.

### 4.2. Intention of Perinatal Nurses to Adopt the BFHI

The theoretical variables were all significantly correlated with* intention* and with each other (Pearson correlation coefficients 0.69–0.94, *P*s < 0.0001). The age of the nurses was the only demographic characteristic correlating with* intention*. The maternity units were also associated to* intention* (chi-square = 77.3; *P* < 0.0001). Following the regression, the variables composing the explicative model of nurses' intention are:* PBC* (*β* = 0.40),* SN* (*β* = 0.32), and* moral norm* (*β* = 0.15). The present study was not planned as a multilevel study allowing testing individuals versus environmental factors. Nonetheless, we have verified if* maternity units* was a factor contributing to explaining nurses' intention. The regression analysis indicates that three sites (2, 3, and 6) were statistically different from the maternity unit of reference. Their respective level of contribution was much lower than 1% of the explained variance. Tests for multicollinearity were performed and none was detected. Variance inflation factors (VIF) were well below 10, the condition index was under 30, and residuals were normally distributed as per Keith [40] recommendations. An analysis of proportion showed that 50.3% of nurses had the intention to adopt the BFHI in their practice, which can be represented by a score ranging from 5 to 7 on the seven-point Likert-type scale used. The final model explained 91% of the variance of the intention of nurses to adopt the BFHI (Table 4).

### 4.3. Perinatal Nurses' Beliefs

The variables retained for analyses were the salient underlying beliefs of the TPB constructs for which a significant relation with* intention* was found. A total of six items representing the underlying beliefs distinguishing those who had a high intention from those who had a low intention were statistically significant. The item “I would be able to adopt the BFHI in my practice despite its strict criteria” explained the greatest portion of the variance (*R* ^2^ = 0.58; *P* < 0.0001). The other items are as follows: “If I adopted the BFHI in my practice, mothers from the maternity units would approve” (*R* ^2^ = 0.57; *P* < 0.0001), “I would adopt the BFHI in my practice if women had an antenatal preparation that is coherent with the BFHI” (*R* ^2^ = 0.30; *P* < 0.0001), “If I adopted the BFHI in my practice, nurses from the maternity units would approve” (*R* ^2^ = 0.09; *P* < 0.001), “I would adopt the BFHI in my practice if there was a lactation consultant in the maternity unit” (*R* ^2^ = 0.07; *P* < 0.001), and “I would be able to adopt the BFHI in my practice, despite the unfavorable remarks of the entourage of the mother on the BFHI criteria” (*R* ^2^ = 0.04; *P* < 0.005).

## 5. Discussion

The aim of this study was to identify the factors influencing the intention of hospital-based perinatal nurses to adopt the BFHI in their practice. Results suggest that the theoretical model contributed to explain a large proportion of variance of nurses' intention. The strongest factors explaining their intention are, respectively,* PBC, subjective norm*, and* moral norm*. PBC is not a one-dimensional construct.* PBC* involves the perceived controllability (perceived freedom) and perceived difficulty (self-efficacy) [41, 42]. Although the participants were not surveyed on Steps 1 and 2, it is possible that their perceived control to adopt the BFHI in their practice was hampered by external factors, regardless of their level of intention. However, when considering the perceived difficulty, high and low intenders differed. Respondents, who believed in their capacity to overcome barriers such as the strict standards of the BFHI, had a stronger intention to adopt it. Previous studies have shown that many nurses discussed the compliance to criteria of the BFHI in terms of dogmatism [9, 17, 43–45].  Table 3 indicates that, while the CODE is implemented in all hospitals, none of them met the standards to successfully implement Step 6. This particular step is frequently reported as being challenging, internationally [4, 17, 20, 46–48].

Nurses can play a major role in preventing in-hospital supplementation that can impede the lactation process, associated with interruption of breastfeeding [14, 49]. The other significant barrier refers to the unfavorable remarks from the mother's support system with respect to nursing practices based on the BFHI. It is well documented that mothers are influenced by significant others [18], but to our knowledge, our study is the first to report that this barrier has an impact which is statistically significant on the nurses' intention to adopt the BFHI. Two facilitating factors underlying* PBC* are statistically significant. Respondents believe that the antenatal preparation provided by the antenatal nurses to pregnant women should be aligned with the BFHI. Nurses' comments that served to form this item of the questionnaire referred to inconsistent nursing advice and a lack of continuity of care in the antenatal period, making their efforts feel useless in hospital settings. Ingram et al. [50] and da Graça et al. [51] underlined the importance for health professionals to provide uniform and accurate messages to women on breastfeeding during their pregnancy. For instance, couples not well prepared to deal with potential obstacles are more likely to supplement early in the process [52]. The antenatal preparation is associated with mothers' breastfeeding initiation [19, 53] and duration [54]. Having a lactation consultant in the maternity unit was identified as the other significant facilitating factor. Considering that only two hospitals offer the WHO/UNICEF 20-hour course, it is plausible that, to adopt the BFHI, many nurses perceive the need to have a lactation consultant in their unit. This perceived need could also be related to the way respondents envision their role and their capacity to influence their entourage. Szucs et al. [55] found that nurses in maternity units avoided fulfilling their counseling role, underestimating their capacity to influence mothers on breastfeeding. Nursing research identifies leadership as a key element to play a counseling role and be influential [56].

*Subjective norm* is the second variable of importance explaining the intention of nurses to adopt the BFHI. This finding indicates that nurses are significantly influenced by others' opinions in their professional practice. In particular, nurses who believe mothers would agree with a nursing practice according to the BFHI standards are more motivated to work accordingly. In their integrative review, Semenic et al. [17] found that a major concern of nurses with respect to mothers' opinion is to be regarded as professionals who impose breastfeeding. The participants are also significantly influenced by the opinion of nursing colleagues. In their critical review on the impact of continuing breastfeeding education, Ward and Byrne [19] reported that nurses valued knowledge and opinions of their peers more than their own nursing education.

*Moral norm* is also a statistically significant variable explaining the intention of nurses to adopt the BFHI, indicating that the BFHI corresponds to their personal values. This finding exposes the dilemma that nurses may experience when they perceive that the opinion of the mothers regarding the BFHI is not aligned with their own values.

Finally, the weak contribution of the organizations in the explicative model suggests that the intention of nurses is mainly determined by individual factors. However, our findings warrant investigation of the organizational factors.

### 5.1. Practical Implications

Our results underline the importance to address the interventions to the entire nursing team when considering the importance of the peer influence. Nurses could benefit from the WHO/UNICEF 20-hour training, which seems to increase their self-efficacy in supporting mothers with breastfeeding [50]. This training is also known to foster a better compliance with the BFHI among nurses [1, 14, 17, 19, 50, 53], among organizations [19] and partners involved in the provision of care in the antenatal period [1]. Nonetheless, not all nurses adopt the BFHI, even with this training [20, 57]. The focus of the WHO/UNICEF 20-hour training is largely oriented on the clinical content. Nurses' involvement in the BFHI may need to be examined in light of clinical leadership skills development as well, as to envision themselves as influential resources. Clinical leadership is defined as “staff nurse behaviors that provide direction and support to clients and the health care team in the delivery of patient care” [58, page  450]. Patrick and collaborators [58] insist that every staff nurse can be a leader whose practices reflect clinical expertise, effective communication, collaboration, coordination, and interpersonal understanding to empower, inform, support decision making, understand various perspectives, network caregivers, mothers and their entourage, and finally, to empathize with others while being connected to their knowledge and clinical judgment to provide judicious care. Clinical leadership training has potential to help nurses to deal with their normative beliefs in order to envision their capacity of influence in a more balanced way, rather than being overly subjected by the influence of others in their practice. Additionally, clinical leadership may help nurses overcome perceived obstacles, such as the strict criteria of the BFHI, which, as recommended by Saadeh [1], should not be “diluted.” Interventions addressing both, BFHI and clinical leadership knowledge and skills, may convey the perinatal nursing practice to a higher level, reducing the need to refer to lactation consultants. This study also underlined the necessity to work in partnership with the antenatal team in order to provide coherent messages to the mothers.

*Study Limitations.* The main limit of this study is its exclusive focus on hospital-based nurses. The adoption of the BFHI is a complex process that goes beyond the hospitals' walls [55] and teams of nurses [1]. It would have been interesting to explore how the implementation of the BFHI in each hospital correlates with the level of intention to adopt the BFHI among their staff and how the measures for the TPB-related constructs differ per institution (and why). However, more organizations and more nurses per organization are needed to conduct a multilevel modeling approach to the data analysis. More research is needed to understand the relationships of organizational measures, such as the implementation of Steps 1 and 2 on nurses' intention to adopt the BFHI and their related professional practices. Finally, these findings should not be generalized to the nurses' population, since the study was conducted among a convenient sample recruited in specific maternity units.

## 6. Conclusions

Breastfeeding is widely recognized for its beneficial impact on human health. The BFHI contributes to increase exclusive breastfeeding and nurses play a crucial role in the successful implementation of the BFHI. However, the adoption of the BFHI can be challenging for nurses. Based on an extended version of the TPB, this study found that* PBC*,* subjective norm,* and* moral norm* explained a large proportion of their intention to adopt the BFHI in their practice. In order to identify the target of actions to increase the proportion of high intenders, we identified the corresponding underlying beliefs of the participants. Nurses are mainly influenced by their perceived capacity to overcome the strict criteria of the BFHI, the mothers' approval of a nursing practice based on the BFHI, and the antenatal preparation of the mothers. Measures such as the WHO/UNICEF 20-hour training combined to the development of clinical leadership skills may contribute to change nurses' perceptions regarding the strict criteria of the BFHI and envision themselves as an influential resource in their professional practice. It is also recommended to work in collaboration with the antenatal teams to foster coherent messages and coordinated care across services. Finally, considering that the achievement of the BFHI involves organizational measures, more research is needed to examine their potential role in the adoption of the BFHI among nurses.

## Figures and Tables

**Table 1:** The ten steps to successful breastfeeding.

**Every facility providing maternity services and care for newborn infants should:**	
(1) Have a written breastfeeding policy that is routinely communicated to all health-care staff	
(2) Train all health-care staff in skills necessary to implement this policy	
(3) Inform all pregnant women about the benefits and management of breastfeeding	
(4) Help mothers initiate breastfeeding within a half-hour of birth	
(5) Show mothers how to breastfeed and how to maintain lactation, even if they should be separated from their infants	
(6) Give newborn infants no food or drink other than breast milk unless medically indicated	
(7) Practice rooming-in: allow mothers and infants to remain together 24 h a day	
(8) Encourage breastfeeding on demand	
(9) Give no artificial teats or pacifiers (also called dummies or soothers) to breastfeeding infants	
(10) Foster the establishment of breastfeeding support groups and refer mothers to them on discharge from the hospital or clinic	

Source: WHO/UNICEF [8].

**Table 2:** Sociodemographic characteristics of the participants.

Sample characteristics (*n* = 159)	Frequency
Mean age (SD)	36.1 ± 11.0 year
Gender	
Female	159 (100%)
Education	
College diploma	98 (61.6%)
University certificate	14 (8.8%)
Nursing degree	44 (27.7%)
Master degree	3 (1.9%)
Experience in perinatal care	
<1 year	11 (7.0%)
1 to 10 years	93 (58.9%)
11 to 20 years	34 (21.5%)
>21 years	20 (12.7%)
Last training on breastfeeding^a^	
<1 year	40 (29.0%)
1 to 5 years	65 (47.1%)
>5 years	33 (23.9%)
Employment status	
Full-time	83 (52.5%)
Part-time	59 (37.4%)
On-call	16 (10.1%)

^a^ *n* = 138; SD = standard deviation.

**Table 3:** Maternity units demographics and BFHI status.

Maternity unit	Birth volume/year	Nurses/maternity unit (FTE^a^)	Lactation consultant in maternity unit	Exclusive breastfeeding at discharge April 1st 2011–March 31st 2012 (%)	BFHI steps implemented	CODE
1	1711	49		No data	2^b^, 5	*✓*
2	787	29.8	*✓*	57.8	1, 2^c^, 3, 4, 5, 6^d^, 7, 8, 9, 10	*✓*
3	538	15.5	*✓*	55.2	1, 2^c^, 3, 5, 6^d^, 7, 8, 9, 10	*✓*
4	277	6		No data	3, 5, 8, 9, 10	*✓*
5	899	28		No data	1, 4, 5, 7, 8, 9, 10	*✓*
6	450	14		No data	3, 4, 5, 6^d^, 9, 10	*✓*

^a^FTE full-time equivalent ^b^2-hour training ^c^21-hour training ^d^partially implemented.

**Table 4:** Final explicative model.

Variables	*β*_standardized_	*P*	*R* ^2^_partial_
Perceived control	0.40	<0.0001	0.77
Subjective norm	0.32	<0.0001	0.10
Moral norm	0.15	0.008	0.04
Maternity centre 2	0.15	0.0002	0.004
Maternity centre 3	0.09	0.0001	0.0008
Maternity centre 4	0.03	0.24	0.0002
Maternity centre 5	0.07	0.14	0.0005
Maternity centre 6	0.08	0.01	0.004

Adjusted *R* ^2^ = 0.91; *F* = 202.42 *P* < 0.0001.
